# Vibration Behaviour of Topologically Optimised Sacrificial Geometries for Precision Machining of Thin-Walled Components

**DOI:** 10.3390/ma19010070

**Published:** 2025-12-24

**Authors:** Evren Yasa, Ozgur Poyraz, Finlay P. C. Parson, Anthony Molyneux, Marie E. Baxter, James Hughes

**Affiliations:** 1 The University of Sheffield Advanced Manufacturing Research Centre North-West, Blackburn BB2 7HP, UKf.p.parson@amrc.co.uk (F.P.C.P.); a.molyneux@amrc.co.uk (A.M.); m.baxter@amrc.co.uk (M.E.B.);; 2 Mechanical Engineering Department, Eskisehir Technical University, 26555 Eskisehir, Turkey

**Keywords:** additive manufacturing, laser powder bed fusion, Ti6Al4V, topology optimisation, tap testing, thin wall machining, vibration

## Abstract

Additive manufacturing (AM) enables the consolidation of components and the integration of new functionalities in metallic parts, but layered fabrication often results in poor surface quality and geometric deviations. Among various surface treatment techniques, machining is often favoured for its capability to enhance not only surface finish but also critical geometric tolerances such as flatness and circularity, in addition to dimensional accuracy. However, machining AM components, particularly thin-walled structures, poses challenges related to unconventional material properties, complex fixturing, and heightened susceptibility to chatter. This study investigates the vibrational behaviour of thin-walled Ti6Al4V components produced via laser powder bed fusion, using a jet-engine compressor blade demonstrator. Four stock envelope designs were evaluated: constant, tapered, and two topologically optimised variants. After fabrication by Laser Powder Bed Fusion, the blades underwent tap testing and subsequent machining to assess changes in modal characteristics. The results show that optimised geometries can enhance modal performance without increasing the volume of the stock material. However, these designs exhibit more pronounced in situ modal changes during machining, due to greater variability in material removal and chip load, which amplifies vibration sensitivity compared to constant or tapered stock designs.

## 1. Introduction

The demand for lightweight structures is increasing across industries such as aviation, aerospace, defence, and automotive. Emerging design approaches, including topology optimisation and implicit lattice structure, enable significant weight reduction while preserving structural performance. However, the successful deployment of such designs in real products ultimately depends on meeting stringent quality requirements. These requirements may relate to material properties, geometric accuracy, dimensional tolerances, and surface integrity, all of which must be controlled to ensure reliable performance and regulatory compliance [1]. Achieving the required level of quality across these characteristics often necessitates the appropriate selection of manufacturing methods, or a combination of them. Among these methods, machining is widely used as a post-processing operation following conventional processes such as forging and casting, as well as non-conventional techniques like additive manufacturing [2]. Despite its widespread use, machining may present several challenges. Certain challenges associated with machining processes can be mitigated through the appropriate selection of process parameters and the application of effective cooling strategies [3]. However, the mitigation of other challenges, such as chatter vibrations, necessitates the utilisation of different approaches. These vibrations during cutting can lead to dimensional inaccuracies and surface defects [4]. These issues become even more pronounced when machining lightweight components with thin-walled or slender geometrical features [5].

Vibration suppression, damping, or minimisation during the machining of thin-walled workpieces is a research topic that has attracted significant interest from both academia and industry [6]. This objective can be addressed through active and/or passive damping techniques [7]. Active damping methods may utilise magnetorheological fluids, piezoelectric actuators, or sound-sensitive magnetic coil systems that apply counteracting forces to suppress vibrations [8,9]. In contrast, passive methods typically involve additional constraints through fixtures or clamps, viscous or phase-change filler materials, or the incorporation of sacrificial geometries to increase structural mass and reduce vibrational response [10,11].

Several concerns may arise when implementing active damping techniques in industrial settings, including harmonic interference resulting from forced vibrations and difficulties in accurately capturing chatter due to ambient environmental noise [9]. Passive damping methods can also present practical challenges. For example, ice-based fixtures may require supplementary cryogenic infrastructure, leading to significant investment and maintenance costs [12]. Additionally, dimensional uncertainties caused by shrinkage and subsequent re-expansion of the machined part must be carefully estimated and compensated for to ensure precision. Alternatively, attaching external masses using viscoelastic tapes or neoprene sheets may pose a risk of mass detachment from the workpiece, particularly under intense or high-pressure cooling conditions [13].

In this context, passive damping techniques that introduce sacrificial geometries can be preferred. While sacrificial geometries can adopt rib- or chord-like shapes [14,15], particularly for filling internal volumes in thin-walled pockets, their application is generally limited to constant or, in some cases, tapered stock materials when dealing with standalone thin-walled structures such as blisks or turbine-blade aerofoils [16]. Tunc and Zatarain (2019) investigated the influence of stock thickness on damping behaviour and observed a reduction in vibration amplitudes as thickness increased [16]. Based on their findings, they identified 2 mm as the optimal stock thickness for the specific blade geometry used in their study.

In another study, Petráček et al. developed a chatter-free machining approach by dividing the blade into several segments and assigning distinct thickness values to each, thereby enabling continuous variation in spindle speed throughout the cutting process [17]. Zhang et al. proposed a non-uniform allowance planning technique for machining thin-walled rectangular blocks, in which the local stiffness at the cutter–workpiece contact point is determined while satisfying constraints on allowable deformation [18]. Karimi and Altintas investigated the use of a stepwise-varying stock during the finishing pass to enhance the rigidity of the semi-finished component [19]. Similarly, Tunc and Gulmez demonstrated that the axial depth of cut can be doubled depending on the cutter’s location, which is directly linked to the in-process shape of the workpiece during machining [20]. Finally, Kienast et al. advanced stock-shape analysis by simultaneously defining multiple stock geometries and cutting locations, performing finite element simulations for each configuration by exporting the in-process workpiece geometry and analysing it in Ansys to capture the evolving state of the machining process [21]. In addition to machining, the improvement of manufacturing thin-walled components using other methods can also be counted among the research trends, and studies in this direction continue with designs and design optimisations [22].

Building upon prior research, this article investigates thin-wall machining in the context of post-additive manufacturing processes, leveraging the design flexibility enabled by techniques such as topology optimisation. In this study, an expanded set of fixed and tapered stock configurations was developed around a jet engine compressor blade demonstrator, extending beyond the designs previously reported in the literature. Furthermore, the feasibility of using additive manufacturing—and its associated design flexibility—was explored. This is achieved through topology optimisation, a technique used to systematically remove unnecessary material from the design domain while satisfying performance objectives. Two topology optimisation studies were conducted to achieve either equivalent frequency or identical stock material mass, based on a selected optimal value within the stock range. These novel geometries, which had not been previously documented in the literature, were then fabricated from a Ti6Al4V alloy using the laser powder bed fusion additive manufacturing technique. The blades were subjected to tap testing, followed by final machining operations, and surface quality measurements.

## 2. Materials and Methods

As outlined in the introduction, the research followed a structured methodology beginning with an extensive literature review to establish the theoretical framework and identify knowledge gaps. The challenges associated with the manufacture and performance of the target components informed the design of demonstrator blades. Material and geometric properties were defined to enable accurate initial finite element (FE) analyses, which were validated through experimental benchmarking.

Parametric studies and topology optimisations were then carried out to refine the designs, supported by surface reconstructions to ensure manufacturability. The optimised geometries were produced via laser powder bed fusion and subsequently heat-treated to improve material properties. Post-processing involved wire electrical discharge machining (WEDM) and tap testing to assess dynamic behaviour.

Based on these results, a down-selection was made for final machining. Throughout the study, modal behaviour and surface quality were monitored to evaluate machining performance.

### 2.1. Designs, Simulations, and Optimisations

**Figure 1:**
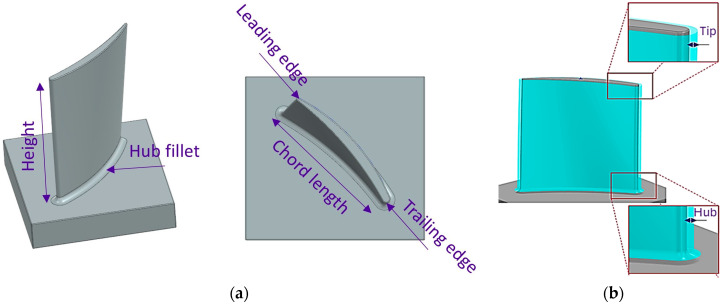
Descriptive legends for blade design variants: (**a**) terms for finished blade features; (**b**) representative stock envelopes at the hub and the tip.

To establish a representative geometry for the research demonstrator, a survey of various commercial and military jet engines was conducted. As compressor blades are typically machined either as individual components or as integral features of a blisk, the compressor module was selected as the reference architecture [23,24]. To standardise the design across engines with differing numbers of stages, the 5th stage was identified as an appropriate average reference point. Accordingly, a 5th-stage compressor blade was defined as the finished demonstrator geometry. The blade has a span of 50.15 mm, a chord length of 42.16 mm, a hub fillet radius of 2 mm, a leading-edge (LE) thickness of 1.25 mm, and a trailing-edge (TE) thickness of 0.5 mm, reflecting typical proportions observed across the surveyed engine designs as shown in Figure 1a. The surface quality of the designed blade was assessed using reflection analysis, in which 256 black and white stripes were projected onto the surface from multiple angles. Distortions in the reflected patterns were used to identify surface irregularities.

Following the completion of the blade design, two conventional stock envelope strategies were employed to prepare the geometry for manufacturing. The first approach applied a constant stock across the entire blade, ensuring a uniform material allowance at both the hub and the tip. The second approach used a linearly tapered stock envelope, reducing the material allowance towards the tip (see Figure 1b). A reference value of 2 mm for the constant stock was selected based on previous findings [16], and this reference was also applied to define two tapered configurations. The first configuration tapered from 3 mm at the hub to 1 mm at the tip, while the second tapered from 2.5 mm to 1.5 mm, resulting in a total thickness equivalent to the constant stock approach. Both the constant stock and the tip thicknesses of the tapered configurations were then reduced in 0.5 mm increments, down to a minimum of 0.5 mm. However, preliminary FE analyses indicated that the 0.5 mm constant stock configuration exhibited excessive vibration amplitudes and significantly reduced natural frequencies as presented in Table 1. Owing to these unfavourable dynamic characteristics, this configuration was excluded from further investigation.

Titanium was selected as the material of choice due to its widespread use in high-performance and safety-critical applications, including biomedical implants, aerospace structures, and turbine engine components [24,25]. To ensure that potential support structures in the PBF-LB process do not negatively affect the flow surface, the blades are manufactured such that their height direction is parallel to the build direction. Consequently, the dominant material properties are expected to align with those of this direction. Still, anisotropy was considered in this work. Before defining the specific material property extraction direction, the difference in properties was examined using the material manufacturer’s catalogues for a layer thickness of 60 µm. According to the catalogues, the difference in the elastic modulus between the build direction and the direction perpendicular to it, for heat-treated specimens, is approximately 6.5% [26]. This difference in the elastic modulus directly affects the material’s elongation value and thus the spring constant (k). However, since the square root of the spring constant is used in frequency calculations, its influence on the modal behaviour will be directly proportional to the square root of the difference, limiting the impact to around 2.5%. Considering the technical situations detailed above, the anisotropy—which results in a very minimal difference—was neglected in this study. This decision aimed at achieving more efficient simulation times across a large number of blade design configurations. The material properties presented in Table 2, which formed the basis for both the FE analyses and topology optimisation (TO), were derived from experimental investigations conducted in prior studies by the University of Sheffield, Advanced Manufacturing Research Centre North West (AMRC NW).

ANSYS Workbench 2024 R2 was employed to perform finite element (FE) analyses and topology optimisations (TO), utilising its integrated harmonic analysis interface to assess the dynamic response characteristics. The FE mesh was constructed using Tet10 elements, which are 10-node quadratic tetrahedral elements. An element edge size of 0.5 mm was chosen to balance computational efficiency with geometric fidelity, and the mesh resolution was set to level 2. A slow transition rate between mesh densities ensured smooth gradation and avoided abrupt changes. Mesh quality metrics indicated robust performance, with an average element quality of 0.85 and a maximum quality of 0.99 on a 0–1 scale, with values closer to 1 indicating more optimal, well-shaped elements. An average quality of 0.85, therefore, suggests that the majority of elements exhibit near-ideal geometry, minimising numerical error and improving solver stability.

**Figure 2:**
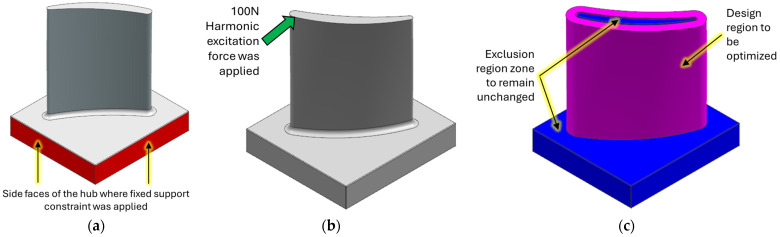
Finite element analyses and topology optimisation descriptions for the blade: (**a**) fixed constraint around the hub; (**b**) harmonic excitation; (**c**) design and exclusion regions.

Harmonic response simulations were conducted to evaluate the blade’s dynamic behaviour. Specifically, the boundary conditions were defined as follows: A fixed support constraint was explicitly applied to the four side surfaces defining the circumference of the blade hub (as shown in Figure 2a). This constraint fixes all translational and rotational degrees of freedom (Ux = Uy = Uz = 0 and Rx = Ry = Rz = 0) on the constrained faces, replicating the real fixture. Excitation was simulated using a 100 N remote force, applied harmonically, at the designated tap-test location (Figure 2b). The vibrational response across the structure was captured.

In the reference configuration, designated V6 in Table 2, the stock envelope had a thickness of 3 mm near the hub and 1 mm near the tip, reflecting representative average values from the literature. For the topology optimisation (TO) process, the geometry was segmented into two distinct bodies: the blade and its surrounding stock envelope. The blade was treated as an exclusion zone to preserve its aerodynamic integrity, while the surrounding envelope was defined as the design domain, allowing material redistribution to enhance performance without compromising the blade’s functional geometry (Figure 2c).

Two optimisation objectives were pursued: the first aimed for mass equivalence with the baseline V6 configuration, ensuring material efficiency and manufacturability; the second targeted matching the vibration frequency of V6, preserving dynamic performance. The TO process was configured with carefully selected parameters to ensure accuracy and structural relevance. A convergence criterion of 0.1% ensured numerical stability, while a penalty factor of 3 enhanced contrast between solid and void regions. To maintain manufacturability and avoid numerical issues, a minimum normalised density of 0.001 was applied, and a 50% density threshold was used to interpret the final material layout, clearly defining regions to retain or remove during post-processing.

**Figure 3:**
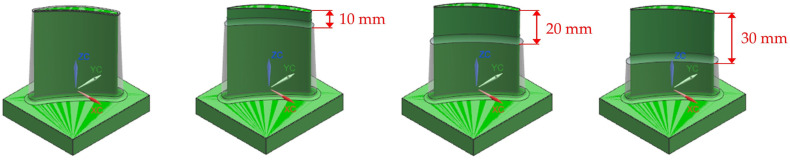
In-process geometries for 10 mm, 20 mm, and 30 mm cutting depths on example V6 (3-1 mm variable stock) blade configuration.

The study aimed to determine the changes in the designed blades and the subsequent topology-optimised configuration not only in their state prior to machining but also in their in-process state. To this end, the method followed by Kienast et al. (2024) [21] was adopted, and in-process geometries were created for each blade in increments of 10 mm up to a depth of 30 mm, which is more than half the blade height. The aforementioned harmonic response simulations were also applied to these geometries. Figure 3 shows the in-process workpieces with cutting location depths of 10 mm, 20 mm, and 30 mm for the exemplary V6 (3-1 mm variable stock) blade configuration.

### 2.2. Laser Powder Bed Fusion and Heat Treatments

Laser powder bed fusion (PBF-LB) was employed using Ti6Al4V powders, with fabrication carried out on a Renishaw AM500Q (Gloucestershire, UK) system equipped with four lasers, each capable of delivering up to 500 W of power at a wavelength of 1070 nm. The laser spot size was maintained at 80 µm, and system calibration was routinely performed by the manufacturer to ensure consistent performance. Builds were conducted on titanium substrate plates with dimensions of 250 × 250 × 35 mm, preheated to 170 °C before the process. Throughout the build, a high-purity argon atmosphere was maintained, with oxygen concentration strictly controlled below 500 ppm to preserve material integrity and prevent oxidation. The material composition is shown in Table 3, whereas the apparent density of the employed powder is measured to be 2.274 g/cm^3^.

During the build of parts, up-skin, down-skin, and blocked paths were turned off as standard. To enhance microstructural uniformity and reduce anisotropy, each successive layer was rotated by 67° relative to the x- or y-axis of the build platform. PBF-LB process was executed using a laser power of 320 W, with a layer thickness of 60 µm, a scan speed of 1500 mm/s, and a hatch distance of 95 µm. These parameters were selected to achieve a volumetric energy density of 37 J/mm^3^, ensuring sufficient energy input for consistent melting, optimal layer bonding, and high-quality part consolidation throughout the build (see Table 4). Using the aforementioned powder, machine, scanning strategies, and process parameters, one sample was fabricated for each of the three blade types: constant stock, variable stock, and topology optimised. These productions encompassed all the down-selected stock dimension configurations.

All fabricated samples were heat-treated in a TAV vacuum furnace Model TH 30/30/30 (Bergamo, Italy) using the manufacturer’s recommended annealing cycle. Specimens were heated from room temperature to 800 °C at a controlled ramp rate of 10 °C/min, held at 800 °C for 240 min, and subsequently furnace-cooled to approximately 100 °C before removal from the chamber. This cycle is intended to relieve residual stresses and stabilise the α/β microstructure by promoting partial decomposition of the as-built martensitic α′ phase, thereby improving ductility and fatigue resistance [27,28]. Temperature consistency across the furnace hot zone is ensured through regular UKAS-accredited Temperature Uniformity Surveys (TUS), and the unit is certified as a Category 5 furnace in accordance with AMS 2750 [29]; in addition, all load thermocouples used during heat treatment are fully calibrated, ensuring that each specimen experienced the intended thermal profile. After cooling, the blades were separated from the build plate by wire electrical discharge machining (WEDM) prior to tap-testing and multi-axis machining operations.

### 2.3. Tap Testing

**Figure 4:**
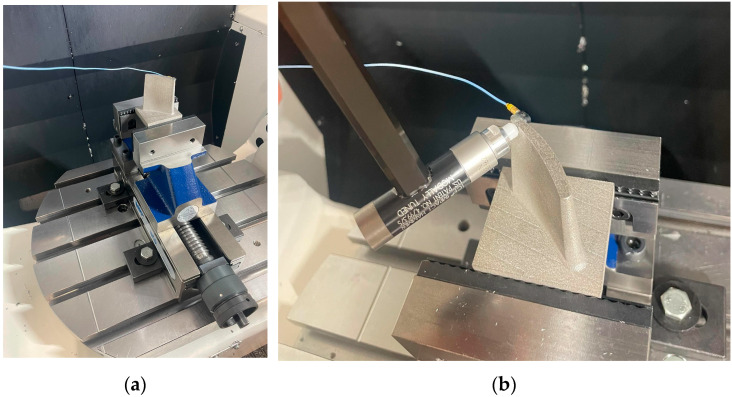
Tap test setup: (**a**) example blade mounted in a vice on the machine table; (**b**) blade tapping action with accelerometer.

The manufactured blades were mounted in a configuration closely replicating their actual machining orientation to ensure realistic testing conditions, as shown in Figure 4a. Tap testing was performed at two critical locations, namely the leading edge and the trailing edge, to evaluate vibrational response. Tap testing, in this context, involved applying a controlled mechanical impulse using an instrumented impact hammer (ICP 50862 hammer, NY, USA) and recording the resulting vibratory response, enabling characterisation of the local dynamic properties and identification of natural frequencies at those locations. An accelerometer (PCB Model 352C23) together with a data acquisition system (ICP Model 485B39) was positioned on the side opposite the tap location to accurately capture the dynamic behaviour (Figure 4b). All components of the system were newly acquired in full calibrated state. To fully characterise the modal response, tests were conducted in both the X and Y directions, providing comprehensive insight into the blade’s vibrational characteristics. The frequency response range was chosen to be 0–5000 Hz, a decision supported by the findings obtained through the preceding finite element analyses. Each location was tested three times, and the average magnitude was recorded. The testing kit was provided by Productive Machines, and its DigiTap software 2025 (Sheffield, UK) was used to visualise results. The visualised results were then exported as comma-separated value (CSV) files and processed in MATLAB R2024 (MA, USA) with average mean smoothing at 10.

### 2.4. Machining and Surface Quality Measurements

Machining was performed on a Mazak Variaxis i-500 (Oguchi, Japan) 5-axis vertical milling machine. The additively manufactured blades were secured using a 5-axis vice with hard-toothed jaws. Three tools were employed: a 10 mm bull-nose end mill for roughing, a 10 mm ball-nose end mill for semi-finishing, and a 3 mm tapered ball-nose end mill for finishing. All tools were held in Sandvik hydraulic membrane holders. A consistent set of cutting parameters was applied across all blades to ensure uniform machining conditions and comparability of results. The finish pass was conducted with a 0.5 mm radial depth of cut and a 0.15 mm axial depth of cut, with spindle speeds chosen based on the stability lobe diagram derived from tap testing with the 3 mm tapered ball-nose end mill, as shown in Table 5.

Toolpaths were created in the Siemens NX 2412 (TX, USA) computer-aided manufacturing (CAM) module and post-processed using the AMRC’s proprietary post-processor. Toolpaths were verified using CGTech Vericut 9.5 (CA, USA) to check for potential collisions with the tooling and workholding setup. Emulsion-based fluids with 9% concentration were used as the coolant for all machining operations.

**Figure 5:**
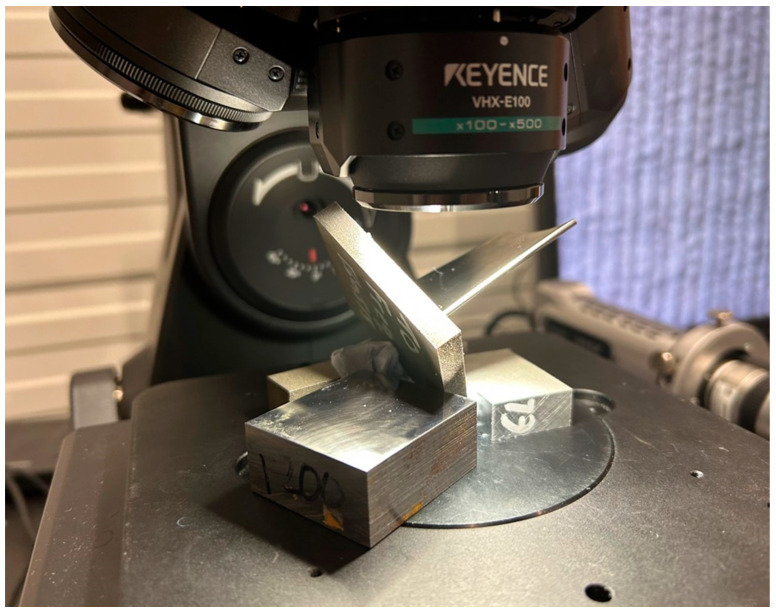
Surface measurement setup of an example blade.

To measure surface quality, as demonstrated in Figure 5, each blade was tilted according to the specific measurement region, such as the hub, tip, leading edge, or trailing edge, to ensure accurate alignment and data capture. Five measurement regions were acquired from each blade face to provide a representative surface profile. Surface quality characterisation was performed using a Keyence VHX-E100 digital microscope (Osaka, Japan) with full-ring illumination to ensure uniform lighting and minimise shadowing effects. Measurements were taken 3 times and at 100× magnification, with each region having a measurement area size of 3 mm × 2.5 mm. The reason for utilising reduced area sizes was to minimize the influence of the freeform surface on the overall surface quality results. The data were subsequently processed using VHX software in 3D depth mode. A whole-plane tilt correction was applied to ensure consistency across angled surfaces and to enable visual interpretation of the surfaces alongside the numerical values obtained. Arithmetical mean height (Sa) of the surfaces was reported without filtering. The fundamental reason for omitting filtering was that tilt correction and planarisation had already been performed for visual examination, and further filtering could potentially reduce the apparent influence of chatter or tool marks.

## 3. Results and Discussions

### 3.1. Simulation and Optimisation Results

**Figure 6:**
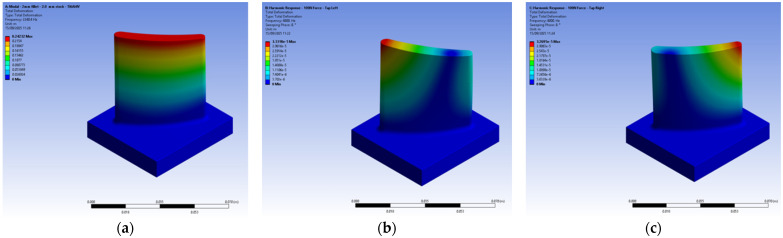
Simulation result examples on C3 (blade with 2 mm constant stock): (**a**) natural frequency; (**b**) harmonic response—force at left side; (**c**) harmonic response—force at right side.

Before proceeding with the analysis and simulations for all design configurations, the frequency values obtained from the analysis were compared with the frequency values obtained from tap testing for the blades with the chosen reference configurations: a 2 mm constant stock envelope and a 3-1 mm variable stock envelope. After confirming that the frequency values remained within a level below 5% divergence following this comparison, the analysis and simulations, as well as the topology optimisations, for all remaining design configurations were completed. Analyses, simulations, and optimisations were conducted successfully and with high computational efficiency, using the specified material properties, mesh configurations, and boundary conditions as illustrated in Figure 6. The results indicate that the modal behaviour of the system can be predicted cost-effectively using these parameters, even in the absence of physical experimentation. This predictive capability is particularly valuable for preliminary stock envelope design. Frequency-domain simulations revealed that the first vibration mode is dominated by bending localised near the blade tip, a finding consistent with previous literature. Under harmonic excitation, regardless of the excitation point, the leading and trailing edge regions consistently exhibited elevated vibration magnitudes, while the central blade region remained relatively stable. This dynamic response pattern aligns with established observations. Budak et al. [30] demonstrated that blade stability is influenced by both the location on the blade surface and the extent of material removal, showing that chatter predominantly occurs near the leading and trailing edges close to the blade tip after two operational steps, corroborating the current simulation outcomes. Karimi and Altintas also investigated an optimal stock removal methodology and found that, irrespective of blade stock type or operational sequence, the natural frequencies consistently decreased in the lower half of the blade [19].

**Figure 7:**
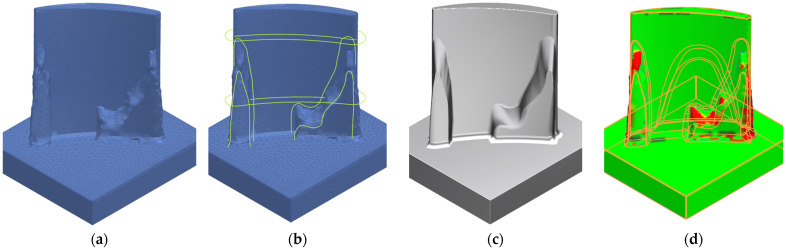
(**a**) Mesh geometry imported into Siemens NX; (**b**) spline curves used in surface reconstruction; (**c**) reconstructed body; (**d**) deviations between mesh and reconstructed surface.

Topology optimisations targeting either mass equivalence (TOM) or frequency equivalence (TOF) with the baseline configuration were performed successfully. However, as TO typically produces material layouts in a raw, mesh-based format—often irregular, noisy, and unsuitable for direct use in CAD/CAM environments—surface reconstruction is essential. This process smooths and refines the geometry, producing a clean, manufacturable shape and enabling its conversion into a CAD model for downstream engineering and fabrication workflows. In this study, the results of the topology optimisations were exported from ANSYS and imported into Siemens NX. Using the reverse engineering module, spline curves were extracted from the mesh geometry, and freeform surfaces were fitted accordingly. The reconstructed geometry was then compared with the original mesh to evaluate and quantify geometric deviations, ensuring fidelity to the optimised design. Deviations between the mesh and reconstructed surfaces were generally maintained within ±0.1 mm, although in some areas, discrepancies reached up to 0.5 mm, as highlighted in red in Figure 7. As the TO method treats each element in the initial mesh as a unit of material, a smaller element size may lead to more detailed and accurate boundary definition in the result. Alternatively, a minimum member size constraint may force the optimiser to maintain a minimum thickness or dimension for any structural element (walls, ribs, corners, etc.). This can prevent the formation of thin, jagged spikes and single-element porous regions. Although employing either method will improve the accuracy of the reconstructed geometry relative to the topology optimisation results, they both carry a significant trade-off in increased solution time and memory requirements.

**Figure 8:**
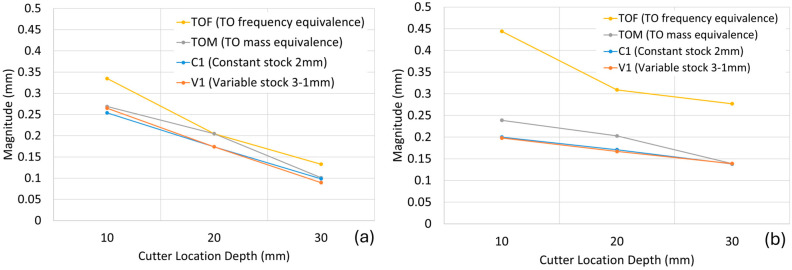
Change in vibration magnitude according to cutter location depth: (**a**) at 1000 Hz frequency; (**b**) at 3000 Hz frequency.

The harmonic analyses for the in-process geometries, which were created with 10 mm cutting depths as specified in the Materials and Methods section, were performed for two selected frequency values and for the fixed, variable, and topology-optimised blades. The obtained results are presented in Figure 8. As can be seen from the figure, regardless of the blade configuration and stock value, a lowering of the cutting depth level—moving away from the tip and closer to the hub—causes the magnitude values to decrease at both frequencies. This behaviour is more linear-like and closer to each other for the blade configurations with fixed (C3) and variable stocks (V6), whereas it is more irregular in the topology-optimised blades (TOF, TOM). This situation can be interpreted by the significant change in the geometric characteristics of the topology-optimised blades as they are machined. Another interesting deduction is related to the blade optimised for equal frequency. This blade lost the similar frequency and magnitude values it exhibited before machining during the first machining operation and showed increased magnitude values compared to the reference blade.

### 3.2. Blade Production and Material Consumption Comparisons

**Figure 9:**
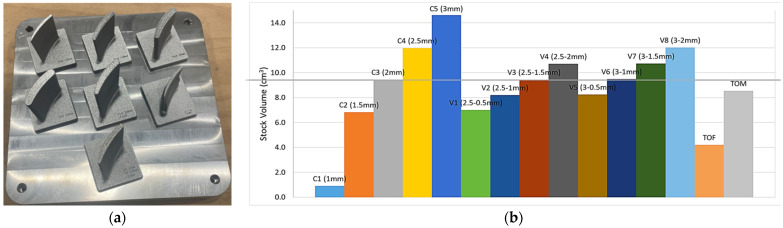
(**a**) Blades following the PBF-LB process; (**b**) bar chart showing stock material volume for each blade configuration.

The laser powder bed fusion process for fabricating the designed and topology-optimised blade geometries was carried out successfully, with no observed issues during production. The blades, manufactured in grouped arrangements on shared base plates, exhibited no signs of discoloration with clearly readable corner markings, as shown in Figure 9a. Post-processing inspection on a granite measurement table confirmed the dimensional integrity of both the base plates and the blades after being separated. Within the selected workflow, including heat treatment and WEDM, dimensional deviations remained within acceptable limits, resulting in minimal distortion and enabling efficient subsequent machining setup and alignment.

The stock material volume consumed during blade fabrication increases proportionally with the stock thickness at the hub and tip. As shown in the stock material usage chart in Figure 9b, the combined stock thickness at these locations serves as a reliable indicator of the total volume used. For example, the C3 blade configuration, with a uniform 2 mm stock at both ends (total 4 mm), results in a consumed volume of approximately 9.5 × 10^3^ mm^3^. This is comparable to configurations V3 (2.5 mm at the hub and 1.5 mm at the tip) and V6 (3 mm at the hub and 1 mm at the tip), both of which also have a cumulative stock thickness of 4 mm. The topologically optimised TOM variant, designed to maintain the same mass while improving vibrational performance, exhibits a volume reduction of approximately 10% compared with the reference V6 blade. This slight discrepancy is attributed to geometric deviations introduced during surface reconstruction, where fitting smooth surfaces onto the irregular mesh led to minor variations in the final blade volume.

### 3.3. Vibration Behaviour of Blades Prior to Machining

Following tap testing with the Productive Machines Impact Hammer kit and initial data visualisation in DigiTap software, the collected data were exported in CSV format for further refinement and analysis. As noted in the finite element analysis section, preliminary simulations had identified significant issues with the 0.5 mm constant stock configuration, which exhibited excessive vibration magnitudes. Accordingly, this configuration was excluded from further study based solely on virtual investigations.

**Figure 10:**
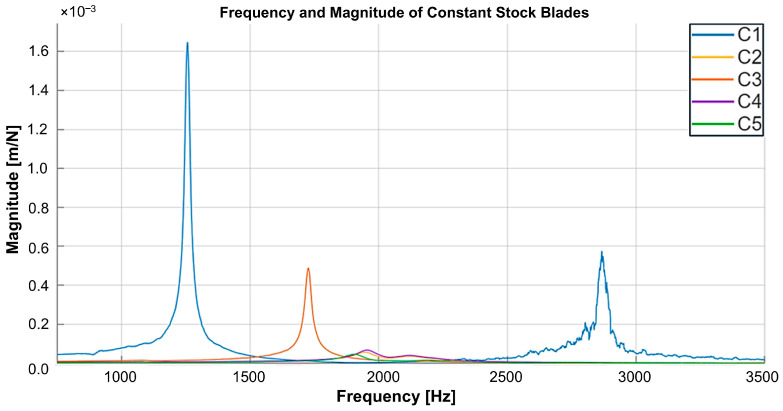
Tap testing results and frequency response of constant stock blades (C1–C5).

Building on this understanding, experimental tap testing was first conducted on constant stock blades to enable a thorough comparative evaluation. The results showed that, in addition to the 0.5 mm stock, blades with 1 mm and 1.5 mm stock thicknesses exhibited significantly lower frequencies and higher vibration magnitudes compared with those having 2 mm or greater stock (Figure 10). This observation aligns with the findings of Tunc and Zatarain [16], who reported that a 1 mm stock thickness corresponds to markedly low absolute stability. Consequently, these thinner stock configurations were excluded from both the benchmarking phase involving variable blades and the subsequent machining trials.

**Figure 11:**
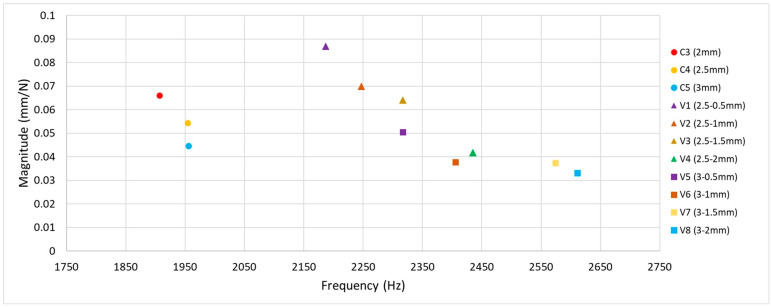
Scatter plot of tap testing results of constant stock blades (C3–C5) and variable stock blades (V1–V8).

Subsequent phases of the research involved cross-benchmarking between constant and variable stock blades to enable a comparative assessment of their dynamic behaviour. Constant stock blades with envelope thicknesses above a certain threshold showed little change in natural frequencies, as their geometric profiles remained largely consistent apart from uniform thickening in the tap testing results presented in Figure 11. In contrast, variable blades with a 3 mm envelope at the hub exhibited higher natural frequencies and lower overall vibration magnitudes compared with those having a 2.5 mm hub envelope. Although increasing the tip stock while maintaining the same hub thickness caused a slight disruption in the trend, it generally resulted in decreased frequencies and increased vibration magnitudes. Notably, for variable blades with a 2.5 mm hub stock, changes in tip thickness could lead to a twofold increase in vibration magnitude, highlighting the sensitivity of dynamic behaviour to tip geometry.

As shown in the stock material usage chart in Figure 9b, the C3 (2 mm) and V6 (3–1 mm) configurations result in identical material consumption. However, the combined tap testing results given in Figure 11 indicate that the variable stock blade exhibits nearly half the vibration magnitude of the constant stock blade, while also achieving a higher natural frequency. This is advantageous, as it avoids coincidence with lower tool rotation frequencies. Benchmarking these designs against optimised blades further highlights performance benefits. The TOF blade, optimised for the same frequency, exhibits a vibration magnitude of 0.1665 mm/N at 2824 Hz. The approximately 10% difference compared with the target can be attributed to deviations observed during surface reconstruction, where fitting smooth surfaces onto irregular mesh data introduced minor discrepancies. Beyond its favourable frequency characteristics, the TOF blade consumes less than half the stock material compared with the C3 (2 mm) and V6 (3–1 mm) configurations, offering a substantial advantage in material efficiency.

### 3.4. Machining Observations and Surface Conditions

Given that the components are made of Ti6Al4V alloy, which can be flammable under certain conditions, a 9% emulsion coolant was employed to ensure safety. However, the use of both external and through-tool coolant delivery generated substantial background noise, making consistent acoustic detection of chatter difficult. Nevertheless, during brief intervals when the coolant flow was temporarily halted for inspection, no signs of chatter were observed.

**Figure 12:**
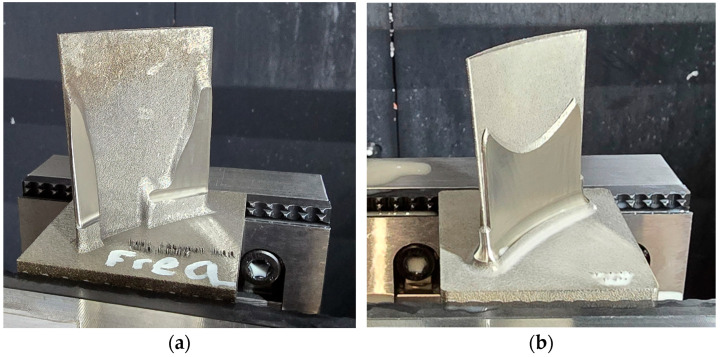
Optimised blades in-process condition between roughing and semi-finishing: (**a**) TOF—topologically optimised blade for the same frequency; (**b**) TOM—topologically optimised blade for the same mass.

Both constant and variable stock blades exhibited a gradual increase in surface texture across the roughing, semi-finishing, and finishing stages, primarily due to the progressive thinning of surrounding stock material. Despite the roughing and semi-finishing passes, the in-process geometries of the optimised blades remained uneven, leading to altered modal behaviour compared with their constant or variable stock counterparts (Figure 12a,b). Furthermore, the evolving shape of the optimised blades resulted in non-uniform stock distribution, introducing variations in the radial depth of cut throughout the machining process. These variations directly affect the material removal rate and chip load, thereby exerting a significant influence on cutting forces [31]. Such cutting force variations may lead to machining instability, particularly during the critical transition between semi-finishing and finishing stages.

Despite the clear advantages of optimised blades, such as higher natural frequencies and reduced vibration amplitudes, their effective utilisation requires careful management. Shape transitions between consecutive cutting stages, such as roughing to finishing, and variations in material removal conditions during machining, must be controlled to maintain process stability and performance. Addressing these challenges may require either a comprehensive design of all machining stages to account for evolving stock conditions or continuous simulation to capture changes in modal behaviour throughout machining. While no commercial software currently integrates both aspects simultaneously, an approach similar to that proposed by Kienast et al. [21], which leverages cutter location data, was adopted in this article to approximate these dynamic effects. Nevertheless, the simulations performed in this article to understand the in-process machining state of the optimised geometries only provide preliminary information. Developing a simulation tool that can solve the geometry optimisation based on modal behaviour coupled with material removal could provide further benefits.

**Figure 13:**
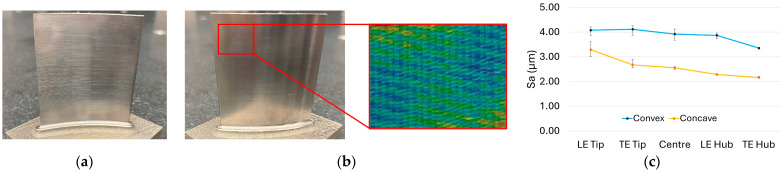
Surface condition of C3 (2 mm constant stock) blade configuration: (**a**) concave face; (**b**) convex face with surface texture mapping; (**c**) S_a_ (µm) surface roughness plot according to different regions of the blade.

Surface quality assessments conducted via microscopy provided valuable insights that complemented the tap testing results. Regardless of blade design or configuration, convex faces consistently exhibited rougher surface textures, evident through both visual inspection and quantitative measurement. This effect is particularly pronounced in the 2 mm constant stock (C3) blade as depicted in Figure 13. Surface roughness was consistently 10–20% higher on convex surfaces than concave surfaces across all blade configurations mainly due to the fact that the outward curvature of convex surfaces leads to higher cutting forces, greater tool deflection, and larger scallop heights during machining, all of which tend to increase surface roughness compared to concave surfaces as pointed out in other studies in the literature [32,33,34].

The distinct surface characteristics of the C3 configuration may be attributed to its uniform stock distribution and relatively stable dynamic behaviour. Compared with thinner, variable, or optimised blades, these consistent conditions make surface differences more readily observable and measurable. Additionally, the C3 blade and other constant stock configurations exhibited a prominent surface roughness gradient, with a noticeable decrease in roughness from the tip toward the hub.

**Figure 14:**
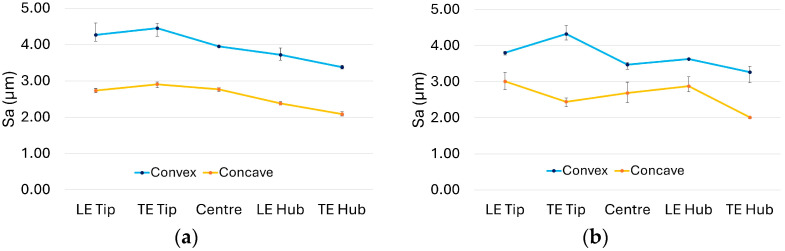
S_a_ (µm) surface roughness plots: (**a**) V6 (hub stock 3 mm the tip stock 1mm); (**b**) TOM—optimised for the same mass.

**Figure 15:**
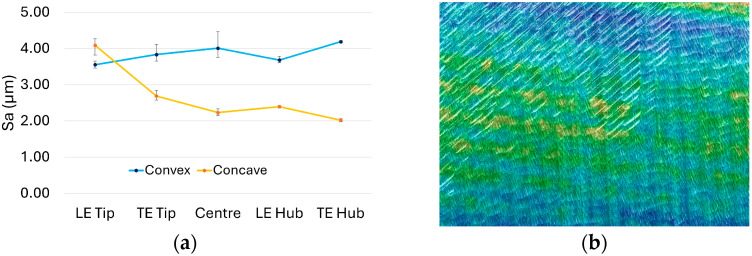
Surface condition of TOF—optimised for the same frequency: (**a**) S_a_ (µm) surface roughness plot; (**b**) surface texture of TOF near the LE Tip.

Representative surface roughness results for selected blade configurations are shown in Figure 14, highlighting key differences without compromising clarity. Specifically, Figure 14a shows a variable blade (V6) with a stock envelope thickness comparable to the constant stock blade illustrated in Figure 13. Figure 14b and Figure 15 depict optimised blade designs, tailored to achieve either equivalent natural frequency or equivalent mass, respectively.

As evident from the images, the variable stock blade exhibits a similarly uniform surface texture to the constant stock blade. In contrast, surface irregularities on both optimised blades are visually pronounced and discernible to the naked eye. These observations are further supported by the S_a_ (µm) surface roughness plots in Figure 14. While the variable blade (Figure 14a) does not show a distinct trend of increasing or decreasing roughness across different regions, its surface texture remains comparatively uniform relative to the optimised configurations. Both optimised blades, however, display pronounced fluctuations in surface roughness along the span from hub to tip (Figure 14b and Figure 15a). These irregularities are likely associated with uneven material removal, variations in radial depth of cut, and the resulting process instabilities.

The surface quality values of the TOF (optimised for the same frequency) configuration, specifically among the other blades, show a loss of the trend between regions. Unlike the other blades, there is no transition from high roughness near the tip to low roughness near the hub. Furthermore, the texture map of the LE Tip region, shown in Figure 15b, is also a significant indicator. While the texture map of the C3 blade configuration in Figure 13b shows regularly distributed peaks and valleys, the texture map of the TOF configuration in Figure 15b exhibits cross-hatched, sharp marks in the upper-left section that would not occur during the material removal by a smooth spherical end mill. These are likely to have occurred during chatter. The resulting surface quality results support the simulation results presented in Figure 8. Especially in the TOF blade configuration, unexpected modal stability problems are felt at a noticeable level during the material removal process.

The preceding research successfully demonstrated a novel approach to stabilising thin-walled blades—initially produced via additive manufacturing (AM)—prior to high-precision, multi-axis milling. By optimising the external stock material (machining envelope) based on the component’s modal characteristics and natural frequency, the blades were rendered inherently more stable for the subsequent machining processes. This methodological innovation led to a significant material saving in the build-up stock, thereby enhancing the overall AM efficiency. However, this geometric optimisation introduced a subtle compromise and trade-off: the complex, optimised geometries resulted in a minor, yet persistent, increase in chatter during certain stages of the milling process, as the instantaneous material removal rate (MRR) fluctuated due to the non-uniform stock distribution. The core trade-off here is between AM material efficiency/reduced build time and milling stability/surface finish quality. Minimising the initial stock (reducing cost/waste) slightly degrades the machining stability (increasing the risk of rework or reduced tool life). In addition to blades, this methodology holds promise for a wide range of thin-walled components such as impellers, nozzle guide vanes, and hollow casings. For certain components, its application may closely resemble the approach demonstrated in the present research, involving modifications to the in-process geometry. However, in cases such as hollow casings, the methodology could extend even further, enabling revisions to the final geometry itself, since these structures inherently incorporate features like bosses and ribs that are integrally connected to the casing walls.

## 4. Conclusions

This study aimed to develop thin-walled additive-manufactured blades with improved dynamic performance while ensuring manufacturability and machining stability. Finite element analysis and topology optimisation were employed to design optimal stock geometries, which were then fabricated using laser powder bed fusion. Tap testing, machining, and surface quality measurements were conducted to evaluate modal behaviour, vibration characteristics, and surface finishes across constant, variable, and optimised blade configurations. The key conclusions from this study are as follows:

- Prior to machining, tap testing was conducted to evaluate the modal behaviour of the blades. The findings demonstrated that optimised geometries can deliver superior modal performance while maintaining the same stock volume to be removed.
- In situ changes in modal characteristics were more pronounced in optimised blades due to variability in material removal rates, radial depth of cut, chip thickness, and cutting forces, compared with constant or variable stock designs.
- Surface roughness was consistently 10–20% higher on convex surfaces than concave surfaces across all blade configurations, primarily because the outward curvature increases cutting forces and tool deflection, as addressed in the literature as contributors to rougher finishes on convex geometries.
- Constant stock blades exhibited a decreasing surface roughness trend from hub to tip; variable stock blades maintained relatively uniform finishes, while optimised blades showed significant surface roughness fluctuations, likely due to uneven stock distribution and process instability.

## Figures and Tables

**Table 1:** Constant and variable stock dimensions of the designed blades.

**Constant**	**C1**	**C2**	**C3**	**C4**	**C5**			
Tip stock (mm)	1	1.5	2	2.5	3			
Hub stock (mm)	1	1.5	2	2.5	3			
**Variable**	**V1**	**V2**	**V3**	**V4**	**V5**	**V6**	**V7**	**V8**
Tip stock (mm)	0.5	1	1.5	2	0.5	1	1.5	2
Hub stock (mm)	2.5	2.5	2.5	2.5	3	3	3	3

**Table 2:** Material properties used for finite element analyses and topology optimisations.

Property	Unit	Value
Density	kg/m^3^	4391
Young’s Modulus	Pa	1.15 × 10^11^
Poisson’s Ratio	-	0.34
Bulk Modulus	Pa	1.97 × 10^11^
Shear Modulus	Pa	4.29 × 10^10^

**Table 3:** Chemical composition of the powders.

Element	Al	Y	V	Ti	O	N	H	Fe	C
Weight (%)	6.02	<0.001	3.93	Bal.	0.10	0.01	0.0010	0.19	<0.01

**Table 4:** PBF-LB scan strategy and process parameters.

Scan Strategy	Parameter	Unit	Value
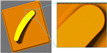	Volumetric Energy Density (VED)	J/mm^3^	37
	Layer thickness	µm	60
	Laser power	W	320
	Scan Speed	mm/s	1500
	Hatch distance	µm	95

**Table 5:** Toolpaths and cutting parameters.

Toolpath	Tool	Operation	Axial Depth of Cut (mm)	Radial Depth of Cut (mm)	Surface Speed (m/min)	Feed Rate (mm/min)
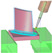	Bull nose—10 mm	Rough	0.25	1	85	0.03
	Ball nose—10 mm	Semi-finish	0.25	0.5	85	0.035
	Tapered ball nose—3 mm	Finish	0.15	0.5	60	0.013

## Data Availability

The original contributions presented in this study are included in the article. Further inquiries can be directed to the corresponding author.
